# First Report of Intentional Intraplaque Lithotripsy After Aggressive Wire Recanalization in Calcified Atheroma and Dilatation (ARCADIA-SHOCK)

**DOI:** 10.1016/j.jscai.2025.104106

**Published:** 2025-12-11

**Authors:** Jeffrey C.Y. Lee, Timothy H.H. Kam, Yasunari Sakamoto, Guangming Tan, Bryan P. Yan

**Affiliations:** a Cardiac Medical Unit, Grantham Hospital, Hong Kong; b Division of Cardiology, Department of Medicine & Therapeutics, Prince of Wales Hospital, Hong Kong; c Department of Cardiology, Toyohashi Heart Centre, Toyohashi, Aichi, Japan; d Division of Cardiology, Department of Medicine & Therapeutics, Faculty of Medicine, The Chinese University of Hong Kong, Hong Kong; e Heart & Vascular Institute, Faculty of Medicine, The Chinese University of Hong Kong, Hong Kong

**Keywords:** case report, eccentric plaque, intravascular lithotripsy, intravascular ultrasound, peripheral arterial disease

## Abstract

Eccentric calcified plaque remains a challenge in the endovascular treatment of peripheral arterial disease despite technological advancements. The "aggressive wire recanalization in calcified atheroma and dilatation" technique was invented to overcome this by intentional intraplaque wiring followed by atherectomy; however, distal embolization can ensue. Substitution with intravascular lithotripsy may be safer and equally effective. To the best of our knowledge, we performed the first case of intentional intraplaque lithotripsy to successfully treat a claudicant patient with heavily calcified bilateral superficial femoral arteries without complications. This may be considered a new technique variation to treat eccentric calcium.

## Introduction

Eccentric calcified plaque poses a significant challenge in the endovascular treatment of peripheral arterial disease (PAD). Even with modern-day equipment such as intravascular lithotripsy (IVL) or atherectomy, vessel preparation can be difficult. Treatment tools are commonly deflected away from the calcium, potentially causing vessel perforation, dissection, or pseudoaneurysm. Besides, atherectomy is associated with slow (or no) flow from distal embolization, which usually necessitates a filter wire.

**Figure 1:**
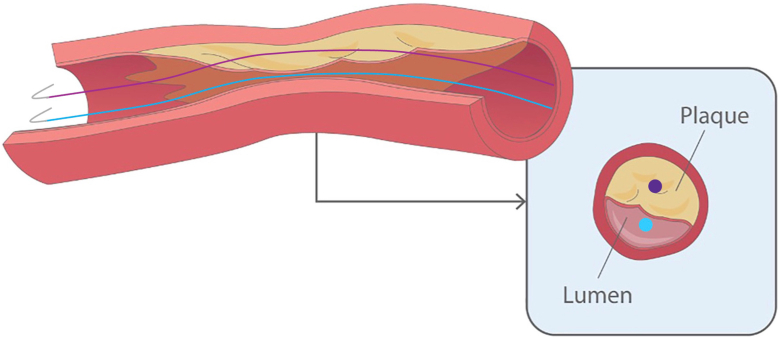
**Schematic representation of the ARCADIA concept. In this diagram, the blue wire (or dot in the short axis view) represents the conventional position in intraluminal wiring.** The wire is only partially in contact with the plaque on one side, making treatment ineffective. In ARCADIA, as denoted by the purple wire/dot, the entire wire is in contact with the plaque circumferentially. This allows much more effective calcium treatment.

Despite these difficulties, lesion preparation is still an important aspect of revascularization, as it translates to better primary patency, less target vessel revascularization, and less restenosis.^1^^,^^2^ More recently, the "aggressive wire recanalization in calcified atheroma and dilatation" (ARCADIA) technique was developed to tackle eccentric calcium.^3^ After intentional intraplaque wiring, our wire becomes centered with reference to the plaque as opposed to the vessel, allowing treatment (usually atherectomy) to be applied more efficiently (Figure 1). To overcome atherectomy-associated distal embolization, we followed ARCADIA with IVL (ARCADIA-SHOCK), which has similar efficacy in calcium modification.^4^ Our case demonstrates the feasibility, safety, and effectiveness of ARCADIA-SHOCK as a variation of the traditional technique.

## Case report

A 64-year-old man with a history of bilateral PAD presented with recurrence of intermittent claudication. Both of his superficial femoral arteries (SFAs) had been treated with drug-coated balloon (DCB) angioplasty 12 months ago, but they remained heavily and eccentrically calcified despite intervention. Left and right ankle-brachial index values were now 0.75 and 0.85, respectively, and pulse volume recording suggested SFA disease. We decided to repeat left SFA angioplasty first, followed by a staged procedure to the right.

Angiogram via right femoral access showed restenosis at the site of previous intervention, namely the distal left and proximal right SFA. A 6F Catapult guide sheath (Boston Scientific) was crossed over to approach the distal left SFA lesion.

**Figure 2:**
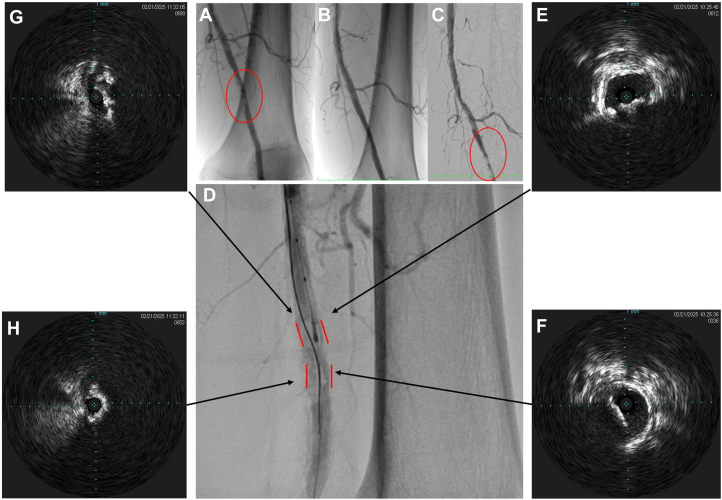
**Fluoroscopy from the index procedure showed heavy calcifications in the distal left SFA, with a residual lesion after angioplasty (A and B).** Digital subtraction angiography (DSA) of the repeat procedure showed the distal SFA calcification more clearly (**C**). Panel (**D**) showed the angiogram of successful ARCADIA wiring, with an intraplaque wire course passing in between the calcium contour (outlined in red). Intravascular ultrasound (IVUS) (Eagle Eye Platinum, Philips) mounted on a 0.014-inch workhorse guidewire in the true lumen confirmed the intraplaque entry of a second Astato XS 20 (Asahi Intecc) wire proximally (**E**) and true lumen re-entry distally (**F**). It also showed the presence of an 180° arc of eccentric calcium approximately 2 to 2.5 mm thick (arrows on **D**). The minimal lumen area (MLA) was 8.5 mm^2^. After wire passage, IVUS mounted on the intraplaque wire demonstrated circumferential calcium (**G** and **H**).

**Figure 3:**
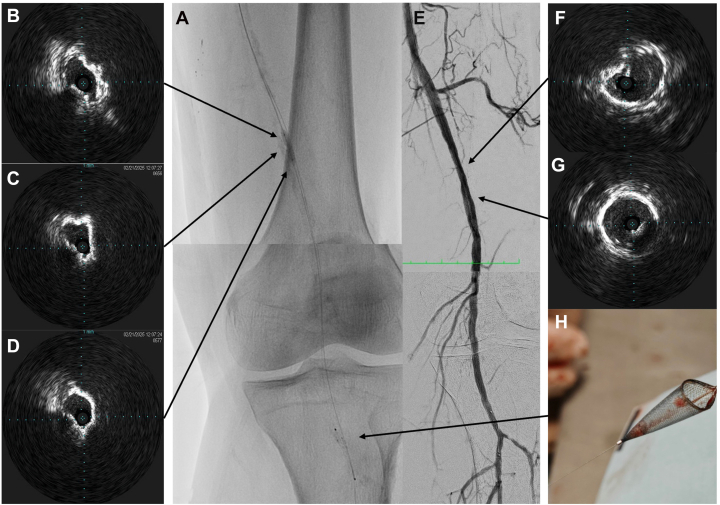
**IVL (Shockwave Medical) was applied to the lesion, with a SpiderFX (Medtronic) distal embolic protection device deployed distally beforehand (A).** Panels (**B**) to (**D**) showed IVUS again from the intraplaque wire. Cracks in the calcium were evident, with an increased luminal gain. DSA showed good results after DCB (**E**). The calcium contour was less prominent at the distal SFA with mild residual angiographic stenosis only. The IVUS (**F** and **G**) also showed a significantly larger lumen. The final MLA was 21.6 mm^2^. The SpiderFX captured a small amount of debris, and there was a good distal outflow (**E** and **H**).

First, we crossed the lesion with a 0.014-inch Glidewire Advantage (Terumo) through the stenotic true lumen. Intravascular ultrasound (IVUS) (Eagle Eye Platinum, Philips) was used to identify the proximal part of the plaque. Using a 0.014-inch Astato XS 20 wire (Asahi Intecc) supported by a 0.014-inch Corsair Armet microcatheter (Asahi Intecc), the calcium was penetrated and wired under fluoroscopic and IVUS guidance. IVUS confirmed a desired intraplaque wire position along with circumferential calcium relative to the wire (Figure 2). Lesion preparation was performed with Shockwave IVL rather than atherectomy as originally described. A 6.0-mm SpiderFX (Medtronic) distal embolic protection device (EPD) was deployed. A 4.0 × 12 mm IVL balloon (Shockwave Medical) was applied to the lesion for 60 pulses, with reassessment IVUS showing satisfactory calcium fracturing. The lesion was further dilated with a 5.0 × 40 mm AngioSculpt balloon (Philips) and a 6.0 × 100 mm Shiden HP balloon (Kaneka Medical). Finally, a 6.0 × 150 mm IN.PACT DCB (Medtronic) was applied with good results (Figure 3). The EPD captured a small amount of debris only. The total procedure time was 120 minutes, and 122 mL of contrast was used.

**Figure 4:**
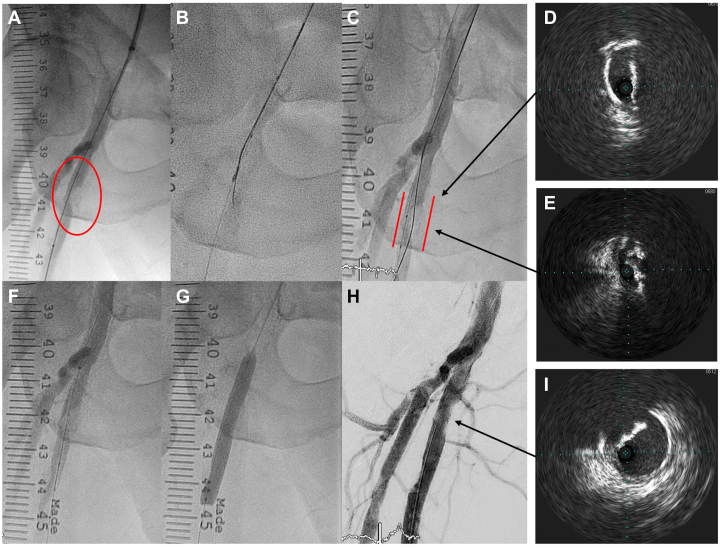
**Angiogram showed severe R SFA stenosis due to eccentric calcium (A).** Wiring through the calcified plaque using the ARCADIA technique was successful (**B** and **C**). Again, panels (**D**) and (**E**) show the IVUS images from both the intraluminal and intraplaque wire. After gentle predilation, the Shockwave M5+ balloon was able to pass through the calcium (**F**). After IVL, the following DCB inflation showed good balloon expansion (**G**), and the final angiogram showed a mild residual stenosis with good flow (**H**). The final IVUS assessment showed no dissection and a large luminal gain (**I**).

The staged right SFA angioplasty was approached in a similar manner (Figure 4). The lesion required escalation to an Astato XS 40 wire to cross, which was again supported by a Corsair Armet microcatheter and IVUS imaging. Predilation was done with a 2.0 × 12 mm balloon, which facilitated the passage and treatment of a 7.0 × 12 mm Shockwave M5+ IVL for 60 pulses. An EPD was not used this time. A 7.0 × 80 mm Ranger DCB (Boston Scientific) was subsequently applied with satisfactory results. There was a good luminal gain and no distal embolization. The procedure time was 96 minutes, and 100 mL of contrast was used.

The patient was prescribed dual antiplatelet therapy for 1 month. At the 1-month follow-up, repeat ankle-brachial index improved to 0.87 (left) and 0.91 (right), with pulse volume recordings showing improved waveforms in both SFAs. He remained claudication-free.

## Discussion and future directions

We demonstrated that ARCADIA-SHOCK is a safe and efficient procedure. Although the eccentric calcified plaques were not severely obstructive, they appear responsible for the patient’s restenosis and repeated symptoms. This warranted a dedicated strategy to ensure more durable results. The ARCADIA technique was first described by Yokota,^3^ which involves intentionally wiring through an eccentric plaque into the distal true lumen. Compared with other advanced calcium-modifying methods (eg, BAMBOO SPEAR),^5^, ^6^, ^7^ ARCADIA is less complex and is more reproducible.

As expected, ARCADIA requires more effort compared with conventional intraluminal wiring. The approach is analogous to that of coronary chronic total occlusions, where microcatheters and IVUS are indispensable. One should have a low threshold to escalate to stiffer wires, as the plaques are often tough and hard. Understanding newer concepts, for example, 3D wiring methods, can also aid success.^8^ The goal is to obtain intraplaque wire position, which turns an eccentric plaque into a “concentric” one and permits more efficient calcium modification. Ideally, predilation with a small balloon would allow a match-sized (to the vessel) IVL catheter to be used, maximizing lumen gain and procedure efficiency (like in our right SFA). Alternatively, using a smaller-sized IVL followed by serial balloon inflations works well too (ie, in the left SFA). Although intraplaque IVL inevitably produces some distal embolization, the degree usually appears insignificant.^4^^,^^9^ The use of an EPD may be considered, and decision-making can be guided by intravascular imaging.

Some potential drawbacks of ARCADIA include vessel dissection and plaque embolization during the wiring process. However, the technique seems to be quite safe. In the original publication, there were no periprocedural complications. In another study where eccentric calcium was treated with an ARCADIA-POBA strategy, there were also no complications.^10^ Other possible limitations include the need for chronic total occlusion skillsets, as well as increased procedure time, contrast, and fluoroscopy use. Nonetheless, these are not prohibitive factors when considering ARCADIA-SHOCK for treating eccentric calcified plaques.

## Conclusion

In summary, ARCADIA-SHOCK is a novel technique in treating eccentric calcium in PAD. After intraplaque wiring, intraplaque lithotripsy appears effective in plaque modification without significant distal embolization. However, more studies and procedures are needed to further validate this technique.

## Declaration of competing interest

The authors declared no potential conflicts of interest with respect to the research, authorship, and/or publication of this article.
